# The expanding phenotype of MELAS caused by the m.3291T > C mutation in the *MT-TL1* gene

**DOI:** 10.1016/j.ymgmr.2016.02.003

**Published:** 2016-02-22

**Authors:** E. Keilland, C.A. Rupar, Asuri N. Prasad, K.Y. Tay, A. Downie, C. Prasad

**Affiliations:** a Department of Pediatrics, Children's Hospital London Health Sciences Centre, London, Ontario, Canada; b Department of Biochemistry, Children's Hospital London Health Sciences Centre, London, Ontario, Canada; c Department of Pathology and Laboratory Medicine, Children's Hospital London Health Sciences Centre, London, Ontario, Canada; d Department of Neurology, Children's Hospital London Health Sciences Centre, London, Ontario, Canada; e Children's Health Research Institute, Children's Hospital London Health Sciences Centre, London, Ontario, Canada; f Medical Imaging, Children's Hospital London Health Sciences Centre, London, Ontario, Canada; g Western University, Children's Hospital London Health Sciences Centre, London, Ontario, Canada; h Paediatric Psychology, Children's Hospital London Health Sciences Centre, London, Ontario, Canada

**Keywords:** MELAS, MELAS 3291T > C, tRNA^leu (UUR)^ mutation, Phenotypic features, Mitochondrial

## Abstract

m.3291T > C mutation in the *MT-TL1* gene has been infrequently encountered in association with mitochondrial myopathy, encephalopathy, lactic acidosis and stroke-like episodes (MELAS), however remains poorly characterized from a clinical perspective. In the following report we describe in detail the phenotypic features, long term follow up (> 7 years) and management in a Caucasian family with MELAS due to the m.3291T > C mutation and review the literature on m.3291T > C mutation. The clinical phenotype in the proposita included overlapping features of MELAS, MERRF (Myoclonic epilepsy and ragged-red fiber syndrome), MNGIE (Mitochondrial neurogastrointestinal encephalopathy), KSS (Kearns-Sayre Syndrome) and CPEO (Chronic progressive external ophthalmoplegia).

## 1 Introduction

MELAS (MIM #540000), most commonly involves an adenine-to-guanine transition mutation at position 3243 of the mitochondrial genome (m.3243A > G) [1]. This mutation involves the *MT-TLI* gene that encodes for a mitochondrial tRNA^leu (UUR)^ [2]. As is true of many of the mitochondrial encephalopathies, there remains a lack of a distinctive genotype-phenotype relationship in MELAS due to heteroplasmy. A rare mitochondrial mutation, m.3291T > C was first identified to be associated with the MELAS phenotype by Goto et al. [3]. Located in the tRNA^Leu (UUR)^, the mutation was described in a Japanese boy with a classic MELAS phenotype [3]. Subsequent reports, however, have identified this mitochondrial mutation in association with a number of different phenotypic presentations (see Table 1). Here, we describe a familial case of a m.3291T > C mitochondrial mutation with MELAS and additional manifestations that have not been previously well characterized.

## 2 Case report

**Fig. 1:**
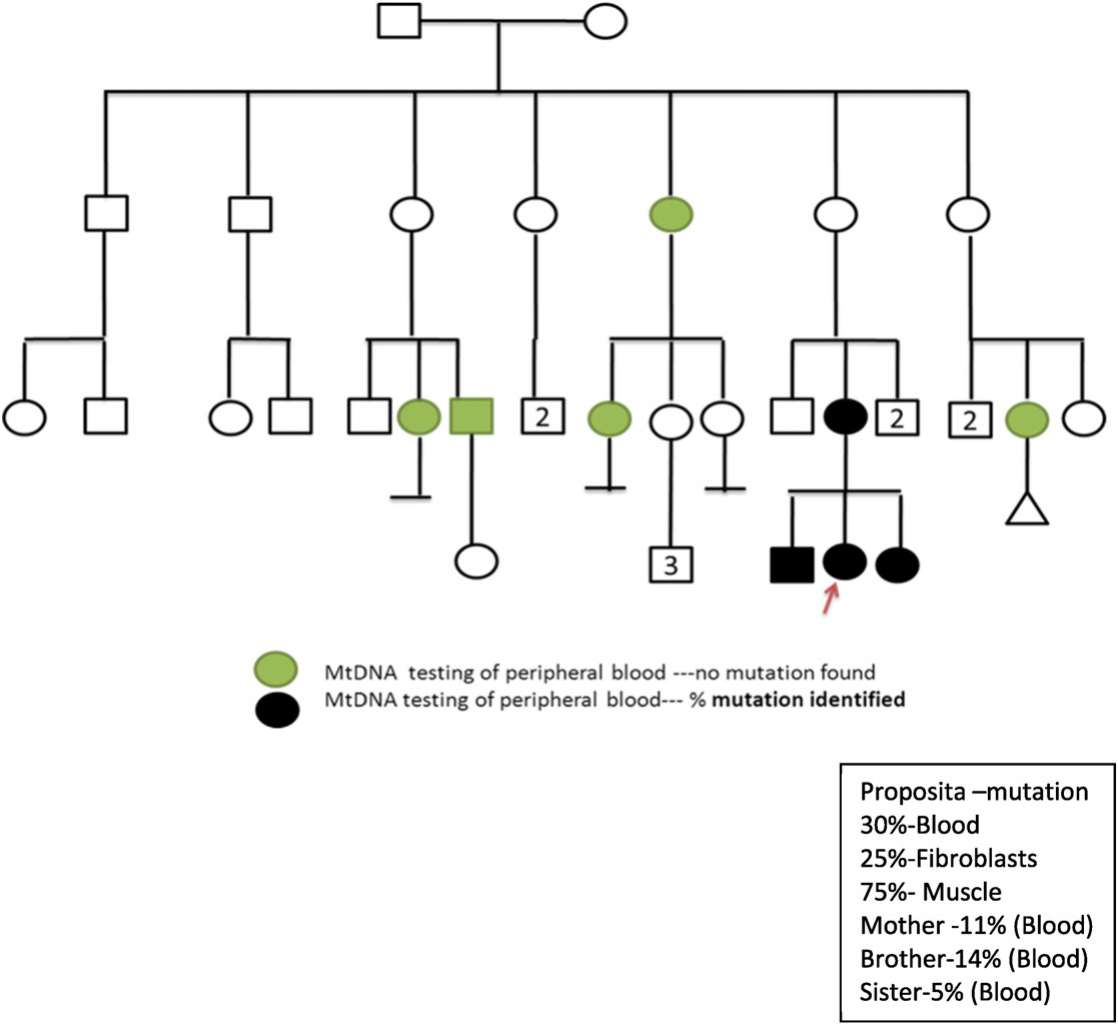
Showing Pedigree with multiple affected individuals.

A 15 year old Caucasian girl of Dutch ancestry was admitted to the pediatric critical care unit for the management of her first episode of status epilepticus. She was the first child of non-consanguineous parents (Fig. 1). Pregnancy and delivery were uneventful and early development was reportedly normal.

**Fig. 2:**
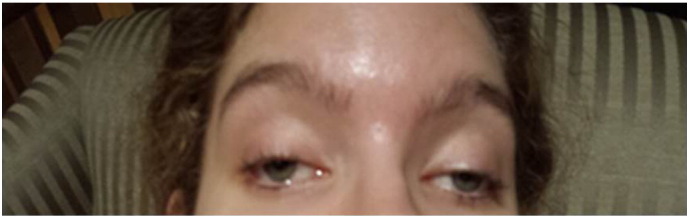
Proposita with bilateral ptosis.

Despite control of seizure activity, during the recovery phase, the patient continued to need ventilation support, and after extubation, remained confused with inconsistent responses, fluctuating awareness and an encephalopathic state. Investigative work up for infectious causes was normal and a urine toxicology screen was found to be negative at admission. Further, on reviewing the history, long standing symptoms of easy fatigability, generalized weakness were noted. Her weight was 35.7 kg (< 3rd centile) and height was 173 cm (75th centile). Neurological examination during recovery disclosed additional clinical findings that included; bilateral ptosis, significant limitation of extra ocular movements (external ophthalmoplegia) (this had been previously missed) (Fig. 2), eyelid myoclonia and distal polymyoclonus in the hands. During the recovery phase and subsequent follow up over the course of a year, recurrent stroke -like episodes, signs of gastroparesis, poor gut motility manifesting with constipation became evident.

The proposita's mother was 36 years old at diagnosis and has suffered from migraine headaches. There was also a history of migraine headaches in the maternal grandmother and a maternal great aunt. The proposita's siblings, an 18 year old brother has recently developed some fatigue and a 16 year old sister has presented with headaches but overall the siblings are relatively asymptomatic. The lactate levels are normal for all the three maternal relatives. The EKG and cardiac evaluation is normal in the mother. The mother is also on the mitochondrial cocktail including riboflavin 400 mg once a day, Carnitor 330 mg 1 tablet 3 times a day, alpha-lipoic acid 100 mg 3 capsules twice daily, CoQ 100 mg 2 capsules 3 times daily, l-arginine 100 mg/mL 60 mL 3 times a day.

### 2.1 Biochemical & Pathology investigations

**Fig. 3:**
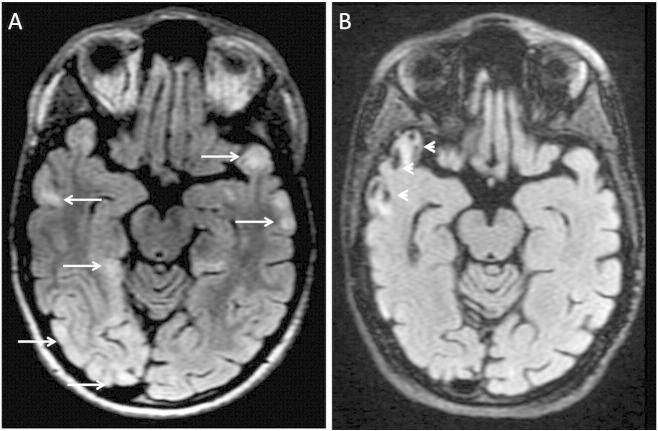
Axial FLAIR (Fluid Attenuation Inversion Recovery, figure A) sequence shows multiple areas of brain parenchymal high signal (long white arrow) in the temporal lobes bilaterally and the right occipital lobe. FLAIR image 4 years later (figure B) reveals an old infarct in the right temporal lobe (white arrowheads) but the other areas of signal abnormalities have returned to normal.

**Fig. 4:**
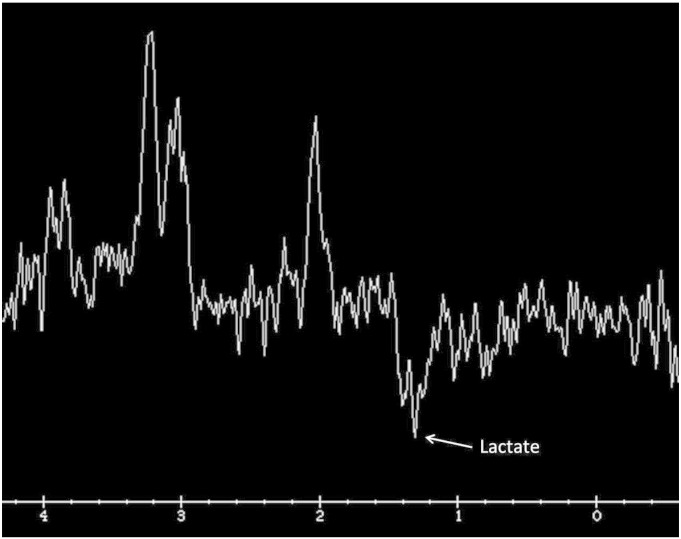
Single voxel MR spectroscopy (TE144) obtained at the same time as figure A showed the characteristic inverted doublet of lactate within one of the areas of abnormality.

Laboratory tests demonstrated a persistent elevation in blood lactate levels 4–7 (normal 0.5–2.2 mmol/L) in the proposita. EEG (Electroencephalogram) confirmed the presence of diffusely slow background rhythms with no epileptiform activity consistent with a moderately severe encephalopathy. Brain magnetic resonance imaging (MRI) showed multifocal ill-defined hyperintense lesions in the grey and white matter of the temporal lobes and orbito-frontal regions (Fig. 3). There was an inverted lactate peak on brain magnetic resonance spectroscopy (MRS) (Fig. 4). A muscle biopsy was performed in the proposita on the left thigh. Histopathology of the stained muscle biopsy of the left thigh showed numerous scattered ragged red fibers which were highlighted by the modified Gomori trichome stain. There were numerous ragged blue fibers positive for succinate dehydrogenase reaction. Many fibers were devoid of cytochrome *c* oxidase (COX). Electron microscopy revealed increased subsarcolemmal accumulation of mitochondria. These mitochondria showed marked variation in size and shape with abnormal cristae architecture. The results of respiratory chain enzyme assays on muscle homogenate were within reference ranges but demonstrated a relatively reduced activity of complex I + III (ratio to citrate synthase 0.041 (0.033–0.110)) in comparison with other complexes. A diagnosis of a mitochondrial cytopathy, likely MELAS was made.

### 2.2 Molecular diagnosis

DNA samples were prepared from the muscle biopsy and blood and urine specimens from the proposita. Sanger sequencing after PCR amplification of tRNA^leu (UUR)^ demonstrated a heteroplasmic m.3291T > C mutation in the proposita. The percent heteroplasmy was calculated using densitometry software (GelDoc-Bio-Rad Image analyzer) after polyacrylamide gel electrophoresis of restriction enzyme (*TSP*509I New England Biolabs, Whitby, Ontario, Canada) fragments after PCR amplification. The level of heteroplasmy varied with tissue sampled: muscle biopsy contained 75% mutated mtDNA, urine 35%, blood 30%, and cultured primary skin fibroblasts 25%. Negative results were obtained with targeted mtDNA analysis for m.3243A > G, m.3260A > G, m.3303C > T, m.8344A > G, m.8993T > G/C and deletions.

The m.3291T > C mutation was also demonstrated in the blood of proposita's mother, brother and sister with 11%, 14% and 5% lymphocyte mutant mtDNA respectively. Urine DNA and other tissues were not tested for the proposita's mother, brother and sister.

The patient is currently in a stable clinical condition and is maintained on a mitochondrial cocktail and anticonvulsants including; Coenzyme Q 120 mg TID, vitamin C 500 mg BID, Thiamin 100 mg OD, Vitamin E 400 IU BID, Creatine 2.5 g BID, lamotrigine 175 mg BID, topiramate 25 mg AM and 50 mg HS and oral *L*-Arginine (0.15 g/kg) TID.

### 2.3 Follow up evaluations

The patient is presently 22 years old and continues to demonstrate ptosis, external ophthalmoplegia, easy fatigability and occasional headaches. Further she has also been diagnosed with Wolff-Parkinson-White syndrome (WPW) on the basis of EKG findings, but remains asymptomatic from a cardiac point of view. She has not had any further hospitalizations since the introduction of arginine to her mitochondrial cocktail. Prior to this she was having at least 2–3 hospitalizations per year.

Initial neuropsychological assessment (15 years of age) was limited due to fatigue, poor attention, distractibility, poor comprehension of task demands, and significant anxiety. She demonstrated weak comprehension of vocabulary (7th percentile) and impaired comprehension of spoken instructions (< 1st percentile) and expressive language (1st–3rd percentiles). Verbal immediate and working memory, and estimates of verbal intellectual and performance (non-verbal) reasoning skills were within the extremely low range (≤ 1st percentile). Performance on a test of verbal learning was average (25th percentile). Although reported to have an academic average over 75% in Grade 9, tests of academic achievement were well below age expectations (< 1st–3rd percentiles). Subsequent assessments carried out at 16 and 17 years of age document improvements in her verbal intellectual skills (10th percentile), understanding of language (13th–30th percentiles), and verbal memory (25th–84th percentile). Impairments in perceptual reasoning (1st percentile), working memory (1st percentile), visual memory (5th–16th percentile), and processing speed (1st percentile) persist with significant difficulty noted in aspects of executive functioning such as cognitive flexibility in day-to-day activities and concept formation (9th percentile). Performance on tests of academic achievement improved but continued to range from just below average to low (3rd–13th percentiles). Currently, she requires guidance and supervision to complete activities of daily living. Due to ongoing fatigue, she completed high school at the pace of one or two subjects at home or in school. She has received counseling in her community for anxiety and helping her cope with the many medical issues and social implications of her diagnosis.

## 3 Discussion

The m.3291T > C mutation is in the TψC loop of tRNA^Leu(UUR)^. The pathogenicity of m.3291T > C has been assessed by several criteria. It is identified in the Mitomap mutations database as causing disease [4]. It is a rare variant that is not present in the Mitomap polymorphism database that is derived from 30,589 panethnic GenBank sequences or the 2704 sequences in the mtDB-Human Mitochondrial Genome Database [5]. m.3291T > C is not highly conserved and 4 (*Cercopithecus aethiops*, *Hylobates lar*, *Macaca sylvanus*, *Papio hamadryas*) of 16 primate tRNA^Leu(UUR)^ with 7 nucleotide long TψC loop is listed in the Mamit-tRNA database [6]. m.3291T > C is replaced by cytosine seemingly without being deleterious. Ding and Leng (2012) questioned the pathogenicity of m.3291T > C mutation on the basis of both the lack of sequence conservation and the predicted secondary structural similarity based on the small change of minimal free energy created by the C > T transition [7]. Kirino et al. [8] demonstrated that the functional impact of the m.3291T > C mutation was to reduce the percentage of the 5-taurinomethyluridine (τm5U) modification at the anticodon wobble position of the tRNA ^Leu(UUR)^ presumably affecting the accuracy or efficiency of translation in common with other MELAS causing mutations. The question of pathogenicity seems to have been resolved by Yarham et al by identifying that the m.3291T > C transition segregated with cytochrome oxidase deficiency in single muscle fibers in a biopsy from a patient who presented with deafness and lipomas [9].

Further support for pathogenicity is the variable heteroplasmy in blood (30%), cultured primary fibroblasts (25%) and muscle biopsy (75%) seen in the patient described herein, the maternal inheritance in this family, the varying level of heteroplasmy and symptoms in the patient's mother and two siblings.

The mutation m.3291T > C was reported in an Italian girl with an isolated myopathy [10], and a young Italian woman with progressive cognitive and behavioral decline with hearing loss [11]. The authors have named this as non-syndromic mitochondrial disorder. Sunami et al described a case of an elderly Japanese woman with the m.3291T > C mutation who presented with mild ophthalmoparesis along with severe cerebellar ataxia, mild proximal myopathy, hearing loss and diffuse paroxysmal slow activity on EEG. There was no lactic acidosis. Clinical manifestations and muscle biopsy suggested MERRF with a family history positive for numerous symptoms suggestive of a mitochondrial encephalopathy [12].

The proposita's clinical presentation, including seizures, stroke-like episodes, recurrent headaches and progressive cognitive decline, along with persistent lactic acidosis and findings on muscle biopsy, WPW syndrome, cranial MRI and MRS are all consistent with the diagnosis of mitochondrial pathology. Based on both clinical and histological findings, initially the proposita's phenotype was deemed consistent with MERRF or CPEO. A similar MERRF/MELAS overlap syndrome due to the m.3291T > C was reported in Chinese teenager [13].

The presence of external ophthalmoplegia is not a common feature of MELAS m.3291T > C and is considered to be typical for KSS or CPEO. One of the recent reports have shown that the m.3291T > C mutation was also described in a man with a MERRF and KSS [14]. Unlike KSS, there was no evidence of ataxia or retinitis pigmentosa in our patient. Our patient did have evidence of cardiac conduction disturbance on EKG showing WPW syndrome; however KSS typically includes prolonged intraventricular conduction time, bundle branch blocks and complete AV block.

**Fig. 5:**
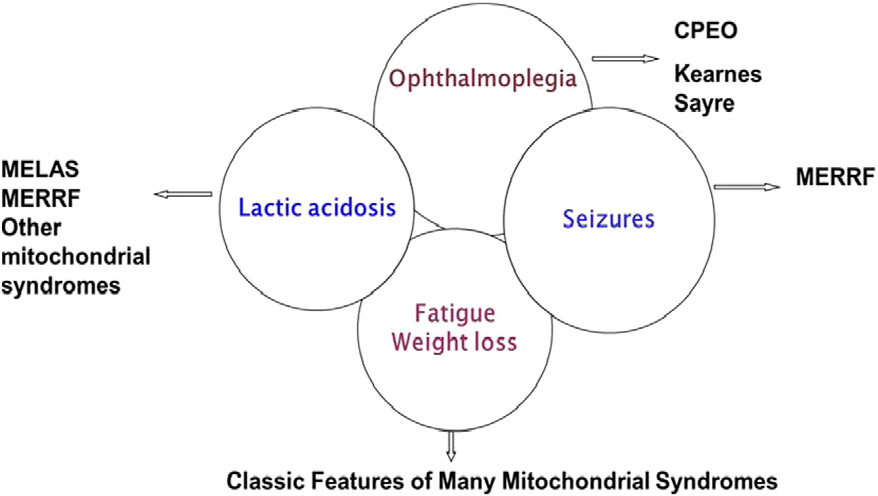
Overlapping Mitochondrial syndromes. (The patient described in the report has many of the features of the clinical phenotypes encountered in MERFF, CPEO, KSS, MELAS, and MNGIE).

Our case is of interest as her presentation is of an overlap syndrome including features of MELAS/MERRF, KSS or CPEO and also MNGIE, poor nutrition and some gastroparesis in association with the m.3291T > C mutation (Fig. 5).

mtDNA mutations demonstrate a large phenotypic variability in their presentation. Similar to m.3243A > G mutation, phenotypes associated with the m.3291T > C mutation are variable. The proposita has responded well to nutrition support and oral arginine [15]. Her current weight is 51 kg. Over the last few years after introduction of oral arginine she has not had any further episodes of status epilepticus or strokes. However more research needs to be conducted on arginine use [15].

## 4 Conclusions

This family extends the phenotype of m.3291T > C mutation to include features of status epilepticus, CPEO/KSS, MNGIE, MERRF and MELAS in the proposita. Based on the above case and the review of literature we propose that the m.3291T > C is a pathogenic mutation. Clinical phenotype will depend on the level of heteroplasmy. Patients with the rare m.3291T > C mutation causing MELAS may also benefit with the addition of arginine to other therapeutic measures.

## Conflict of interest

None.

## Abbreviations

MELAS mitochondrial myopathy: encephalopathy lactic acidosis and stroke-like episodes
MERRF: myoclonic epilepsy and ragged-red fiber syndrome
MNGIE: mitochondrial neurogastrointestinal encephalopathy
tRNA: transfer RNA
CPEO: chronic progressive external ophthalmoplegia
KSS: Kearns–Sayre Syndrome
RRF: ragged-red fiber
WPW: Wolff–Parkinson–White syndrome
AV: block atrioventricular block

## Figures and Tables

**Table 1:** Clinical phenotypes reported with m.3291T > C mutation.

Age (yrs)	Gender	Clinical features	Lactate level (0.5–2.2 mmol/L)	Other laboratory investigations	Molecular investigations	Mitochondrial syndrome	Reference
19	M	Cerebellar ataxia Myoclonic seizures Stroke like episodes Migraine	17.1	Myopathic EMG EEG Multiple spike and wave complexes CT scan bilateral symmetric calcification in basal ganglia RRF in muscle	m.3291T > C 93% in muscle	MELAS/ MERRF	[13]
70	F	Cerebellar ataxia Myopathy Headaches Myoclonus Epilepsy Bilateral hearing loss	Normal	Myopathic EMG EEG diffuse slowing MRI atrophy or frontal lobe and cerebellar cortex RRF in muscle	m.3291T > C 16-27% in blood amongst various family members	Mitochondrial encephalo-myopathy	[12]
11	F	Cerebellar ataxia Seizures Stroke like episodes Migraine	Not known	MRI cerebral infarction EEG slow wave dysrhythmia RRF in muscle	m.3291T > C 86% in muscle 30% in whole blood	MELAS	[3]
23	F	Progressive cognitive and behavioral decline Cerebellar ataxia Migraine Hearing loss	2046 μmol/L (500–1800)	MRI Diffuse supratentorial and infratentorial atrophy EEG slow wave dysrhythmia EKG WPW syndrome RRF in muscle	m.3291T > C 95% in muscle	Non-syndromic mitochondrial disorder	[11]
7.6	F	Mild myopathy	(4238 mM, <1800)	MRI Diffuse supratentorial and infratentorial atrophy EEG slow wave dysrhythmia EKG WPW syndrome RRF in muscle	m.3291T > C 87% in muscle	Mild myopathy disorder	[10]
48	M	Complex phenotype of myoclonus epilepsy with ragged-red fibers (MERRF) syndrome and Kearns-Sayre syndrome (KSS): progressive myoclonus epilepsy, cerebellar ataxia, hearing loss, myopathic weakness, ophthalmo-paresis, pigmentary retinopathy, bifascicular heart block	24.1 mg/dL (normal < 22)	Muscle biopsy demonstrated markedly increased RRF	m.3291T > C 92% mutant in muscle	(MERRF) syndrome and Kearns-Sayre syndrome (KSS)	[14]
